# Butyphthalide in the treatment of massive Cerebral Infarction

**DOI:** 10.12669/pjms.35.1.320

**Published:** 2019

**Authors:** Xinmin Wang, Yingjun Sun, Shugang Dong, Xiaoying Liu, Jinming Ji

**Affiliations:** 1 Xinmin Wang, Department of Neurology, Binzhou People’s Hospital, Binzhou, 256610, China; 2 Yingjun Sun, Department of Endocrinology, Binzhou People’s Hospital, Binzhou, 256610, China; 3 Shugang Dong, Department of Neurology, Binzhou People’s Hospital, Binzhou, 256610, China; 4 Xiaoying Liu, Department of Neurology, Binzhou People’s Hospital, Binzhou, 256610, China; 5 Jinming Ji, Department of Neurology, Binzhou People’s Hospital, Binzhou, 256610, China

**Keywords:** Massive cerebral infarction, Butyphthalide, Curative effect

## Abstract

**Objective:**

To evaluate the effect of butyphthalide in the treatment of massive cerebral infarction.

**Methods:**

One hundred and twenty patients with massive cerebral infarction who were admitted to the hospital between January 2017 and December 2017 were selected and divided into a treatment group (n = 60) and a control group (n = 60) using random number table, 80 each group. Patients in the control group were given conventional cerebral infarction therapy, while patients in the treatment group were given butyphthalide injection besides the conventional treatment. The National Institutes of Health Stroke Scale (NIHSS) score, score of activity of daily living (ADL), lipoprotein-associated phospholipase A2 (LP-PLA2) and prognosis were recorded and compared between the two groups. The response rates of the two groups were recorded.

**Results:**

The total response rates of the control group and treatment group were 73.85% and 93.85% respectively at the postoperative 21^st^ day, and the difference had statistical significance (P<0.05). The NIHSS score of the two groups obviously decreased, and the ADL score significantly increased after treatment; the differences of NIHSS score and ADL score before and after treatment in the same group had statistical significance (P<0.05). The improvement of the indexes of the treatment group was obviously superior to that of the control group, and the differences between the two groups had statistical significance (P<0.05). The level of LP-PLA2 of both groups significantly decreased at the postoperative 21^st^ day, and the difference before and after treatment in the same group was statistically significant (P<0.05); the treatment group had a significantly lower level of LP-PLA2 than the control group, and the difference had statistical significance (P<0.05). The treatment group had significantly higher positive outcome rate and lower mortality rate than the control group at the postoperative 90^th^ day, and the differences had statistical significance (P<0.05). The incidence of adverse events of the treatment group and control group was 8.3% (5/60) and 5.0% (3/60) respectively, suggesting no significant difference (P>0.05).

**Conclusion:**

Butyphthalide has a favourable effect in treating massive cerebral infarction. It can repair neurologic impairment, improve activity of daily living, and adjust the level of LP-PLA2, suggesting favourable application values.

## INTRODUCTION

Massive cerebral infarction refers to ischemic necrosis of cerebral tissues induced by complete occlusion of the main cerebral artery. Its incidence is 10 %~20% that of cerebral infarction. It has extremely high fatality rate and disability rate and poor prognosis due to the sudden onset, high severity and high complexity.^1^,^2^ Currently there are no unified diagnostic criteria in China as well as overseas. Most foreign investigators considered diameter of infarction larger than 3.0 cm, infarction area involving more than 2 lobes or infarction area not smaller than 20 cm^2^ or larger than 2/3 of the ipsilateral hemisphere as massive cerebral infarction,^3^ while infarction area larger than 3/5 of the middle cerebral arterial dominance region or 4/5 of the anterior cerebral arterial dominance region was defined as massive cerebral infarction in China.^4^

Effective therapies for cerebral infarction are limited. Though thrombolytic therapy is the most effective therapy for acute cerebral infarction, but due to the narrow treatment time window, only few patients can accept venous thrombolytic therapy.^5^ Patients who are attacked by malignant middle cerebral arterial infarction within 48 hour, ages below 60 years, and had severe increased intracranial pressure can be rescued by decompressive craniectomy, but it has a low clinical application rate because of high incidences of middle and severe disabilities after surgery and high surgical risks.^6^,^7^ Butyphthalide is a novel drug whose independent intellectual property rights are owned by China. Some studies have found that butyphthalide was effective and safe in the treatment of acute cerebral infarction,^8^ but few clinical studies relate to its efficacy in the treatment of massive cerebral infarction.^9^

In this study, the efficacy of butylphthalide and sodium chloride injection and butylphthalide soft capsules in the treatment of massive cerebral infarction was evaluated, aiming to explore new effective therapies for massive cerebral infarction.

## METHODS

One hundred and twenty patients with massive cerebral infarction who were admitted to our hospital between January 2017 and December 2017 were selected and grouped into a treatment group and a control group using random number table. All the patients signed informed consent. Moreover the study protocol was approved by the ethical committee of our hospital.

Patients who suffered from cerebral infarction less than 12 hour before, satisfied the Guidelines for the Prevention and Treatment of Cerebrovascular Disease (2010),^10^ aged between 18 years and 80 years, had 0~1 point of modified Rankin Scale (mRS) score before onset, confirmed as anterior circulation massive cerebral infarction by cerebral computed tomography (CT) or magnetic resonance imaging (MRI), and did not undergo intravenous thrombolysis and endovascular treatment before grouping were included. But those who had severe heart diseases, liver, renal and pulmonary insufficiency, immune system disease, tumors, posterior circulation infarction, unstable vital signs, or mental diseases were excluded.

Patients in the control group were given conventional treatment including anti-platelet aggregation, lipid regulation, plaque stabilization, oxygen free radicals elimination and intracranial pressure reduction; the treatment lasted for 7 to 14 days. Patients in the treatment group were given 100 mL of butylphthalide and sodium chloride injection which was composed of 25 mg of butylphthalide and 0.9% sodium chloride (batch no.: 618130705; CSPC NBP Pharmaceutical Co., Ltd., China), twice each day, for 14 days, and sequential oral administration of butylphthalide soft capsules (batch no.: 118130712, CSPC NBP Pharmaceutical Co., Ltd., China; 0.1 g/pill), 0.2 g each time, thrice each day, for seven days.

### Evaluation criteria for clinical effect

The curative effect was divided into five grades according to NIHSS scoring standards and the Guidelines for the Diagnosis and Treatment of Acute Ischemic Cerebral Stroke of China (2010). Patients were evaluated as basically cured if NIHSS score decreased 91%~100% and disability was at grade 0. The disease condition was considered having significant progress if NIHSS score decreased 46%~90% and disability was at grade 1~3. The disease condition was considered having a progress if NIHSS`1 score decreased 18%~45%. The disease condition was evaluated having no changes if NIHSS score decreased or increased 17% or lower. Cerebral infarction was considered aggravated if NIHSS increased 18% or higher. The computational formula for the overall response rate was overall response rate =(number of basically cured cases+number of cases of significant progress+number of cases of progress)/total number of cases.

### Observation of indexes

**1)*NIHSS:*** Neurologic impairment was evaluated using NIHSS before treatment and at the postoperative 21^st^ d. NIHSS content included consciousness, contemplate, field of view, upper limb movement, lower limb movement, ataxia, sensation, language, dysarthria and unilateral neglect; the total score was 30 points. Higher NIHSS score indicated severer neurologic impairment.^11^
**2)*Activity of daily living (ADL):*** The improvement of daily living was evaluated using modified Barthel indexes (MBI) including food intake, ornament, bath, dressing, controlling urination, controlling defecation, toilet use, transfer of bed and chair, movement and walking up and down stairs; the total score was 100 points. Higher ADL score indicated better ability of daily living.^12^
**3)*Level of lipoprotein-associated phospholipase A2 (LP-PLA2):*** Fasting elbow venous blood was collected before treatment and at postoperative 21^st^ day. The level of LP-PLA2 in the venous blood was measured using enzyme-linked immunosorbent assay (ELISA). Different reagents were prepared using kits (Tianjin Kangerke Biotechnology Co., Ltd., China) following the instructions on the knit. The absorbance was taken as the vertical coordinate, and the concentration was taken as the horizontal coordinate; then curves were drawn. The level of LP-PLA2 was obtained according to the absorbance value and standard curve, and < 175 ng/mL was considered as normal.
**4)*The modified RANKIN scale (mRS) score at the postoperative 90^th^ day:*** good outcome was given 0 ~ 1 point of mRS score.
**5)**Blood, urine and stool routine indicators, coagulation indicators, liver and renal function indicators, electrocardiograph condition and cerebral computed tomography (CT) or Magnetic Resonance Imaging (MRI) results between the admission and the postoperative 90^th^ day were recorded. The medication related adverse events were also detected and recorded.

### Statistical methods

Data were statistically analyzed using SPSS version 22.0. Measurement data were expressed as mean ± standard deviation (SD). Two independent sample t-tests were used in the comparison between groups. Enumeration data were expressed as rate (%) and processed by Chi-square test. P<0.05 indicated that difference was statistically significant.

## RESULTS

One hundred and twenty patients with massive cerebral infarction were included in the study, and each group included 60 patients. None of them were lost to follow up or quit. The differences of baseline data between the two groups had no statistical significance (Table-I).

**Table-I:** General data between the two groups

General data	Control group(n=60)	Treatment group(n=60)	X^2^/t value	P
Age (year)	63.787±10.31	64.31±8.15	1.753	>0.05
Male n(%)	39(65.0)	41(68.3)	0.075	>0.05
Smoking n(%)	23(38.3)	24(40.0)	0.073	>0.05
Hyperlipemia n(%)	29(48.3)	31(51.7)	0.068	>0.05
Hypertension n(%)	31(51.7)	30(50.0)	0.068	>0.05
Auricular fibrillation n(%)	7(11.7)	6(10.0)	0.259	>0.05
Diabetesn (%)	25(41.7)	28(46.7)	0.137	>0.05
Coronary atherosclerotic heart disease n(%)	27(45.0)	26(43.3)	0.286	>0.05
History of stroke n(%)	45(75.0)	44(73.3)	0.091	>0.05
NIHSS score (point)	18.15±2.77	18.22±1.85	0.486	>0.05

**Fig.1:**
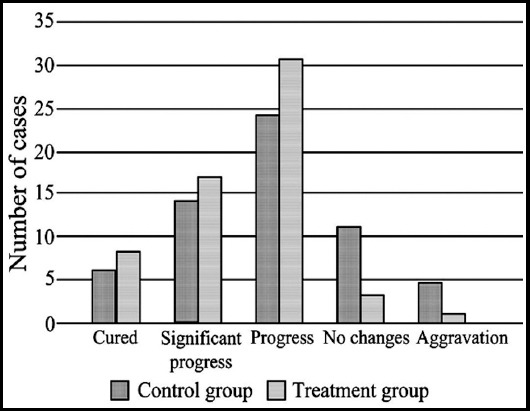
Clinical effect between the two groups.

In the control group, there were six cured cases, 14 cases of significant progress and 24 cases of progress; the overall response rate was 73.3%. In the treatment group, there were eight cured cases, 17 cases of significant improvement and 31 cases had improvement; the overall response rate was 93.3%. The difference of the overall response rate between the two groups was statistically significant (P<0.05; Fig.1).

NIHSS scores of both groups significantly decreased, and ADL scores increased significantly after treatment; the difference of the same group before and after treatment was statistically significant (P<0.05). The improvement of the NIHS score and ASL score of the treatment group was obviously superior to that of the control group, suggesting a statistically significant difference (P<0.05; Table-II).

**Table-II:** NIHSS score and ADL score between the two groups (mean ± SD)

Group	Time point	NIHSS score (point)	ADL score (point)
Control group	Before treatment	18.15±2.77	26.09±4.18
	Postoperative 21^st^ day	10.62±4.28^*^	48.47±4.83^*^
Treatment group	Before treatment	18.22±1.85	27.63±6.42
	Postoperative 21^st^ day	5.58±3.81^*#^	60.24±5.65^*#^

Note:

* indicated P<0.05 in the comparison of the same group before treatment;

# indicated P<0.05 in the comparison with the control group after treatment.

It was found at the 21st postoperative day that the level of LP-PLA2 of both groups significantly decreased, and the difference before and after treatment in the same group had statistical significance (P<0.05). Moreover the treatment group had large decrease than the control group, and the difference was statistically significant (P<0.05) (Table-III).

**Table-III:** Level of LP-PLA2 between the two groups (mean ± SD)

Group	Time point	LP-PLA2 (ng/mL)
Control group	Before treatment	202.18±12.09
	Postoperative 21^st^ day	161.47±13.24
Treatment group	Before treatment	204.59±11.26
	Postoperative 21^st^ day	143.86±11.15

The proportion of patients with good outcome, i.e., 0 ~ 1 point of mRS score, in the treatment group and control group was 45.0% (27/60) and 15.0% (9/60) respectively at the postoperative 90^th^ day; the treatment group was significantly higher (x^2^=8.047, P<0.05). The death rate of the treatment group and control group was 1.7% (1/60) and 18.3% (11/60) respectively at the postoperative 90^th^ day, suggesting a notable difference (x^2^=5.472, P<0.05).

### Analysis of safety

The relevant laboratory indicators and other adverse events were recorded before and after treatment. There were no significant changes in laboratory indicators such as blood, urine routine and tool routine indicators, renal function indicators, blood lipid and blood glucose in the two groups before and after treatment, and allergic symptoms such as allergic dermatitis and rash were not found. The incidence of adverse events in the treatment group and the control group was 8.3% (5/60) and 5.0% (3/60), respectively, with no significant difference (x^2^= 1.471, P>0.05). The incidence of elevated serum aminotransferase was 5.0% (3/60) and 3.3% (2/60) respectively, with no significant difference between the two groups. The observed adverse events in the treatment group also included electrocardiographic changes (one case) and nausea and vomiting (one case), and the difference with the control group had no statistical significance.

## DISCUSSION

Massive cerebral infarction which is featured by sudden onset, severe illness and high fatality rate is a difficulty faced by neurologists. Efficient drugs which are effective in improving the prognosis of massive cerebral infarction have not been found yet. After the occurrence of massive cerebral infarction, cerebral perfusion sharply decreases, and central necrotic zone and surrounding ischemic penumbra appear. A large amount of oxygen free radicals are released after ischemia and hypoxia of cerebral tissues, which induces intracellular calcium overloading, mitochondrial energy metabolism impairment and excitatory amino acid toxicity and leads to nerve cell damage.^13^,^14^ Therefore early recovery of blood perfusion in ischemic brain tissues and promoting the establishment of collateral circulation of ischemic penumbra are keys in the treatment of massive cerebral infarction, and moreover strengthening cerebral nerve protection is also needed.

Butyphthalide, a novel drug independently developed by China, has been extensively applied in the treatment of nervous system diseases such as ischemic cerebrovascular disease and dementia. Butyphthalide may protect nerves through inhibiting cell apoptosis by reducing caspase enzymes activation, inhibiting release of cytochrome-c and down-regulating Fas protein expression, protecting damaged neurons by restraining inflammatory reactions, inhibiting delayed neuronal death by eliminating free radicals, relieving neuron damages by inhibiting calcium overloading, alleviating neurotoxicity by reducing release of excitatory amino acid, controlling encephaledema by protecting blood brain barrier and improving microcirculation of cerebral ischemia area and energy metabolism of brain cells.^15^-^19^ Animal pharmacodynamics study suggested that butyphthalide could block multiple pathological links of cerebral injury induced by ischemic cerebral stroke, narrow infarction area of local cerebral ischemia of rats, relieve encephaledema, improve cerebral energy metabolism and microcirculation and blood flow in cerebral ischemic region, inhibit apoptosis of nerve cells, and resist cerebral thrombosis and anti-platelet aggregation. The results of this study demonstrated that the NIHSS and ADL scores of patients in both groups improved after treatment, the improvement of NIHSS and ADL scores of the treatment group was significantly superior to that of the control group, and the overall response rate of the treatment group was higher than that of the control group, indicating that butyphthalide could enhance prognosis, reduce mortality, and improve long-term activities of daily living in the treatment of massive cerebral infarction.

LP-PLA2 is a specific marker which is generated by macrophages and can reflect vascular inflammation. It plays an important role in the oxidation of Low Density Lipoprotein (LDL), the occurrence and development of atherosclerosis and the occurrence of coronary heart disease and stroke. Elevated level of LP-PLA2 is an independent risk factor in the prediction of the risk of coronary heart disease and stroke.^20^,^21^ LP-PLA2 which belongs to the enlarged phospholipase A2 super family is a serine lipase composed of 441 amino acid residues. It has been known that LP-PLA has 2 types: plasma-type LP-PLA2 and cell-type LP-PLA2. Its coding gene (PLA2 G7) was cloned for the first time in 1995. About 80% of PLA2-PLA2 binds with LDL. LP-PLA2 is mainly produced by inflammatory cells, and the pro-inflammatory products catalyzed by LP-PLA2 involve all stages of atherosclerosis and ischemic cardio-cerebrovascular disease.^22^ Determination of plasma LP-PLA2 level may serve as an independent risk factor for early warning of the risk of ischemic stroke and coronary heart disease.^23^ The results showed that the level of LP-PLA2 in both groups improved after treatment, and the improvement in the treatment group was more obvious than that in the control group, suggesting that butylphthalide treatment could significantly reduce the level of LP-PLA2 and was beneficial to clinical outcomes.

In this study, the proportion of patients with good outcome in the treatment group was significantly higher than that in the control group at the postoperative 90^th^ day, and the mortality rate of the treatment group was significantly lower than that in the control group. A multi-center, randomized, double-blind clinical study carried out butylphthalide injection sequential therapy for 14 days and butylphthalide soft capsule treatment for 90 days;^24^ the results showed that butylphthalide treatment group had a significantly better mRS score than the positive control group at the postoperative 90^th^ day, supporting the results of this study. It is concluded that butylphthalide can improve the prognosis of patients with massive cerebral infarction, reduce mortality, and improve the long-term self-care ability of patients. A previous trial found that the major adverse reaction of butylphthalide treatment was mild or moderate aminotransferase elevation.^25^ In this study, transaminase elevation was also observed in 5.0% of the patients treated with butylphthalide, and there was no significant difference between the two groups. This proportion was significantly lower than that in a study of Zhu et al.^26^, which was considered because of the small sample size of this study In addition, no other obvious adverse reactions were recorded, indicating that butylphthalide was relatively safe in the treatment of massive cerebral infarction.

## CONCLUSION

Butylphthalide has apparent efficacy in the treatment of massive cerebral infarction. Butylphthalide can effectively relieve the nerve function injury, improve daily living ability, and adjust LP-PLA2 and has a high safety. It is worth clinical application and promotion. However, the sample size in this study was small, and the patients were not followed up for a long time. More cases should be included in the subsequent study, and middle or long-term follow up is also needed. Moreover the safety and effectiveness of butylphthalide in the treatment of massive cerebral infarction needs to be analyzed more comprehensively.

### Authors’ Contribution

**XMW, YJS, SGD & XYL:** Study design, data collection and analysis.

**JMJ:** Manuscript preparation, drafting and revising.

**XMW:** Review and final approval of manuscript.
